# Implementation of Game-based Oral Health Education *vs* Conventional Oral Health Education on Children’s Oral Health-related Knowledge and Oral Hygiene Status

**DOI:** 10.5005/jp-journals-10005-1446

**Published:** 2017-02-27

**Authors:** Azhar Malik, Sumit Sabharwal, Aina Kumar, Praveen Singh Samant, Abishek Singh, Vineet Kumar Pandey

**Affiliations:** 1 Associate Professor, Department of Conservative Dentistry and Endodontics, Indira Gandhi Government Dental College, Jammu, Jammu and Kashmir, India; 2 Senior Lecturer, Department of Conservative Dentistry and Endodontics, Seema Dental College and Hospital, Virbhadra Road, Pashulok Post Rishikesh, Uttarakhand, India; 3 Senior Lecturer, Department of Conservative Dentistry and Endodontics, BR Ambedkar Institute of Dental Sciences & Hospital, Patna Bihar, India; 4 Professor and Head, Department of Conservative Dentistry and Endodontics Saraswati Dental College and Hospital, Lucknow, Uttar Pradesh India; 5 Postgraduate Student (Final Year), Department of Conservative Dentistry and Endodontics, Sardar Patel Post Graduate Institute of Dental and Medical Sciences Lucknow, Uttar Pradesh, India; 6 Postgraduate Student (1st Year), Department of Conservative Dentistry and Endodontics Saraswati Dental College and Hospital, Lucknow, Uttar Pradesh India

**Keywords:** Game-based oral health education, Oral health knowledge, Plaque scores.

## Abstract

**Introduction:**

For the prevention of oral health problems, health education of schoolchildren has a vital role. The oral health status of the children can be improved if health promotion in schools is conducted in a comprehensive and interesting manner

**Objective:**

Effectivity of game-based oral health education over conventional on the oral health-related knowledge and oral hygiene status among 8- to 12-year-old schoolchildren.

**Materials and methods:**

A total of 150 children aged 8 to 12 years were divided into two groups. A pretest evaluation of their knowledge regarding oral health and the estimation of plaque index was carried out. Children in group I were given oral health education through PowerPoint presentation once daily for 7 days. Children in group II were educated through the play method (i.e., crosswords and quiz with PowerPoint presentation). The evaluations regarding oral health-related knowledge and plaque scores were recorded on postintervention 1 and 3 months.

**Results:**

In group II, high knowledge scores of 10.32 and 9.98 were obtained by the on postintervention 1 and 3 months respectively. In both the groups, there was a significant increase in good oral hygiene scores and a significant decrease in plaque scores on postintervention 1 and 3 months follow-up, but much better scores were seen in group II compared to group I at both the follow-ups.

**Conclusion:**

Implementation of crossword game-based oral health education program is an easy and effective aid for teaching oral health instructions and preventing oral diseases in children as the knowledge scores of children increased considerably when the game-based teaching intervention was used.

**How to cite this article:**

Malik A, Sabharwal S, Kumar A, Samant PS, Singh A, Pandey VK. Implementation of Game-based Oral Health Education *vs* Conventional Oral Health Education on Children’s Oral Health-related Knowledge and Oral Hygiene Status. Int J Clin Pediatr Dent 2017;10(3):257-260.

## INTRODUCTION

Oral health is integral to general health; it has been rightly said that one is not healthy without good oral health.^12^ One of the key factors in maintaining good oral health is the knowledge of proper oral hygiene practices. Such knowledge given in the formative years of child will be there for lifetime. School age is the formative period physically as well as mentally, transforming the school-child into a promising adult. Health habits formed at this stage will be carried to adult age, old age, and even to next generation. Thus, for the prevention of oral health problems, health education of schoolchildren has a vital role.^3^ It is not only important to teach young children the guidelines regarding brushing and role of diet in oral health interventions but also to develop their interest towards learning habits for a lifetime of good oral health for which school-based health promoting programs are the ideal ways.^4^ In most of these programs, traditional health education aids, such as lectures, demonstration, and models are used which are proven to have a minimal or short-term effect on children.^5,6^ Moreover, studies have also shown that such type of programs have relatively little effect on the cleanliness of the children’s teeth.^7-11^ The oral health status of the children might be improved if health promotion in schools is conducted in a comprehensive and interesting manner.^12^ To make the process of learning a joyful activity for children, there should be well integration of education and entertainment,^13^ which can be done by using various cost-effective media and materials. The innovative educational mode of teaching can be game-based teaching which can have dual effect of facilitating and reinforcing child’s learning in a thought-provoking and self-motivating format. It can be one of the choices for teaching basics of health.^14^ Literature search supports the effect of game-based intervention programs in teaching general health concepts, but there is a dearth of literature focused on oral hygiene intervention. Rising number of dental problems in the country makes such innovative preventive strategy to commence which is the need of the hour. Thus, present study was undertaken to compare the effectiveness of conventional (PowerPoint presentations) and game-based teaching (quiz and crossword puzzles combined with PowerPoint presentations) on the level of oral health knowledge and oral health status among 8- to 12-year-old schoolchildren.

## MATERIALS AND METHODS

The study protocol was reviewed and approved by the Institutional Review Board of a private dental institute. Members from the research team had made first round contact with the authorities in the selected school, and were explained about the purpose, aim, and nature of the study, including children over there. Written consent was obtained from the parents of all the participating children. The study was conducted in an elementary school in Lucknow, Uttar Pradesh, India. The school had 419 children aged 8 to 12 years. Around 150 children aged 8 to 12 years were randomly selected for the study using a computer-generated table of random numbers. Of the 150 children, 75 were randomly assigned to group I and 75 to group II. A 15-item closed-ended semi-structured questionnaire was designed to assess the level of knowledge regarding the oral hygiene of these schoolchildren. Prior to the start of the study, the questionnaire was tested on 50 study subjects. The questions were underwent subsequent revisions before the main study for the understanding of subjects. Cronbach’s alpha and split-half reliability values were 0.81 and 0.88 respectively. Only the reliability and validity was assessed from the pilot study and these subjects were not included in the main study.

The proforma consisted of demographic data and information regarding children’s knowledge on oral health, brushing, and diet. Correct answers for knowledge questions were given a score of "1" and wrong answers were given a score of "0." Four examiners who were trained and calibrated before the start of the study carried out the clinical examination. Interexaminer reliability was 0.79%. Once the interview was completed, the subject’s teeth were disclosed using AlphaPlac Two Tone disclosing agent for examination of plaque index. Six surfaces of all the teeth present, i.e., mesiofacial/buccal, midfacial/buccal, distofacial/buccal, mesiolingual, midlingual, distolingual were scored for subject’s dental plaque scores by Turesky, Gilmore, Glickman modification of the Quigley-Hein index.^15^

The baseline clinical examination was made before the school meal in the mornings in a mobile dental clinic with a standard dental chair and light, within the school premises. The intervention was started after the pretest evaluation of their knowledge regarding oral health and estimation of plaque scores. The calibrated dental examiners gave a 15 minutes lecture on oral health, brushing, and diet using PowerPoint presentation. About 75 children in group I were given oral health instruction through the PowerPoint presentation once a day for 7 days. Around 75 children in group II were instructed using the game-based teaching method (crosswords and quizzes) combined with PowerPoint.

The investigator explained the rules for the game and they were allowed to play once a day for a week. The evaluations regarding plaque scores and knowledge were recorded on postintervention 1 and 3 months follow-up. The resulting data were coded and analyzed to assess intergroup differences. The unpaired and paired t-test (SPSS version 15) was used to evaluate inter and intra group differences respectively between the variables; p ≤ 0.05 was considered statistically significant.

## RESULTS

In group II on postintervention, the oral health knowledge score dramatically increased from 4.40 to 10.32 and for group I scores increased from 4.46 to 8.32 at 1 month (Table 1). Table 2 also shows the follow-up data collected 3 months postintervention. The mean oral health knowledge scores were 7.94 and 9.98 for groups I and II respectively. The lower scores in 3 months postinterven-tion indicates less retention of knowledge over a period of time, but still the scores were higher than baseline and more knowledge retention was seen in group II. Statistically, significant improvement in the plaque scores of the children after postintervention was seen in both the groups but clinically much lower scores were seen in group II compared to group I at both the follow-ups (0.90 and 1.26) (Table 3). There was no significant difference between plaque scores at 1 and 3 months in both the groups (Table 4).

**Table 1:** **Table 1:** Comparison of mean knowledge between two groups at various time intervals by using unpaired t-test

		*Group*		*n*		*Mean*		*Std deviation*		*p-value*	
Baseline		I		75		4.46		0.99		0.94	
		II		75		4.40		0.99			
1 month		I		75		8.32		0.84		0.001*	
		II		75		10.24		0.89			
3 months		I		75		7.94		0.91		0.001*	
		II		75		9.98		0.82			

*p < 0.05, significant

**Table 2:** **Table 2:** Comparison of mean knowledge within the two groups at various time intervals by using paired t-test

*Groups*				*n*		*Mean*		*Std* *deviation*		*Mean difference*			
I		Baseline		75		4.46		0.99		3.86		0.001*	
		1 month		75		8.32		0.84					
		Baseline		75		4.46		0.99		3.48		0.001*	
		3 months		75		7.94		0.91					
		1 month		75		8.32		0.84		0.38		0.06	
		3 months		75		7.94		0.91					
II		Baseline		75		4.40		0.99		5.84		0.001*	
		1 month		75		10.24		0.89					
		Baseline		75		4.40		0.99		5.58		0.001*	
		3 months		75		9.98		0.82					
		1 month		75		10.24		0.89		0.26		0.07	
		3 months		75		9.98		0.82					

*p < 0.05, significant

**Table 3:** **Table 3:** Comparison of mean plaque scores between two groups at various time intervals by using unpaired t-test

		*Groups*		*n*		*Mean*		*Std deviation*		*p-value*	
Baseline		I		75		3.04		0.79		0.94	
		II		75		3.07		0.58			
1 month		I		75		1.23		0.43		0.001*	
		II		75		0.90		0.32			
3 months		I		75		1.55		0.35		0.001*	
		II		75		1.26		0.51			

*p < 0.05, significant

**Table 4:** **Table 4:** Comparison of mean plaque scores within the two groups at various time intervals by using paired t-test

*Groups*				*n*		*Mean*		*Std* *deviation*		*Mean difference*			
I		Baseline		75		3.04		0.79		1.81		0.01*	
		1 month		75		1.23		0.43					
		Baseline		75		3.04		0.79		1.49		0.01*	
		3 months		75		1.55		0.35					
		1 month		75		1.23		0.43		0.38		0.083	
		3 months		75		1.55		0.35					
II		Baseline		75		3.07		0.58		2.17		0.01*	
		1 month		75		0.90		0.32					
		Baseline		75		3.07		0.58		1.81		0.01*	
		3 months		75		1.26		0.51					
		1 month		75		0.90		0.32		0.36		0.076	
		3 months		75		1.26		0.51					

*p < 0.05, significant

## DISCUSSION

Games are desirable learning mode, as they can make studying more entertaining and have been widely utilized for study by students well as teachers, across all age groups and areas of education.^16-18^ Game-based health education approach implemented in the classroom have numerous benefits, it is a powerful method of teaching students to adopt thought provoking means of studying. It helps students to seek reasons for good and bad health. It motivates pupil to understand and learn the facts about health rather than only memorizing, thereby improving cognitive development and building confidence.^19^ This study is game-based oral health intervention associated with the reinforcement of messages on oral health knowledge through crosswords and quizzes. Although most schools encourage health programs with professional awareness on the subject, very few provide the proper emphasis on oral health educational interventions (McDonald and Avery, 1994). The programs in almost all schools usually provide a great deal of information in a short time period and fail to consider several important aspects required to improve oral health habits. Studies found that the retention of complicated information is better with varied learning techniques which stimulate verbal or visual stimuli, resulting in best retention and process of information.^20^ The use of various teaching aids not only acclimatizes a range of learning styles, but also minimizes tedium in the classroom.^16^ Due to the versatility and flexibility of crosswords, they have been used successfully in many different disciplines.^21^ The already known familiarity with crosswords reduces the need to explain directions, saving class time.^17,18^ Additionally, these puzzles are often perceived as being a recreational activity, and thus observed as less threatening and more enjoyable than traditional teaching techniques.^18,21,22^ Game-based health education can be a mainstay for identification of weaknesses in terms of difficulty in understanding, problems in comprehension, and areas to be dealt more intensively etc.^16,17,21^ Studies have also revealed that these puzzles increase students’ interest and motivation in the topic.^16,18^ Using games that include health and hygiene messages can be an alternative for teaching basic health concepts.^14^ In this study, a game-based intervention program that relied on visual stimuli among children in group II that helped them grasp oral health instructions easily, such as good dental hygiene and dietary habits. There was even better quality memorization as well retention of these instructions for a long period of time which was reflected as significantly increase in knowledge scores and a significant reduction of plaque scores calculated at 1 and 3 months follow-up. A statistically significant reduction in the plaque scores was obtained in this study. This finding was in agreement with a study conducted by Swain et al, which showed that implementing school-based contingency dental hygiene resulted in significant reductions in the plaque scores. Improved plaque scores were sustained over a 3 month period proving the effects of game-based learning persisted over time. The game-based educational cum interventional program proved very effective in improving the oral health knowledge and practice of the children in group II as compared to PowerPoint-based oral health interventional program that involved didactic learning. The game-based teaching can meet the criteria of utilizing entertaining, easy-to-understand, and practical educational material recommended for the oral health program. Thus, the crosswords game with oral health instructions can be an effective interventional aid for teaching basic oral health concepts to the children and motivating children for better oral health leading to lower plaque scores.

The results of the study should be interpreted in the light of limitation as children were chosen from one school only and students have the tendency to discuss what all they have learned, this might dilute the exact information regarding oral health knowledge. Nonetheless, the study provides a stage for incorporation of game-based learning about oral health in schools.

## CONCLUSION

This study demonstrated that implementation of crossword game-based oral health education program is an easy and cost-effective method for teaching oral health instructions and preventing oral diseases in children. Future research is needed to further explore differing impacts of demographics, level of study in school, gender, and academic skill (measured by exam scores) on child’s learning and oral health status for a better understanding of implementing of game-based learning in schools.
